# Phenotype of patients with congenital adrenal hyperplasia due to 11β-hydroxylase deficiency

**DOI:** 10.1186/1687-9856-2015-S1-P52

**Published:** 2015-04-28

**Authors:** Vu Chi Dung, Nguyen Phuong Mai, Nguyen Huy Hoang, Bui Phuong Thao, Nguyen Ngoc Khanh, Can Thi Bich Ngoc, Nguyen Phu Dat

**Affiliations:** 1 National Hospital of Pediatrics, Hanoi, Vietnam; 2 Vietnam Academy of Science and Technology, Hanoi, Vietnam

## 

Congenital adrenal hyperplasia (CAH) is one of the most common metabolic diseases. It is caused by a severe or partial impairment of adrenal steroidogenesis affecting cortisol biosynthesis. Approximately 5–8% of all cases are due to steroid 11β-hydroxylase deficiency (11OHD; OMIM +202010), which occurs in approximately 1:100,000 to 1:200,000 live births in non consanguineous populations. Mutations in the CYP11B1 gene, causing 11b-hydroxylase deficiency in the zona fasciculate in the adrenal cortex, have been identified. Our aim is to describe clinical and biochemical features in patients with CAH due to 11β-hydroxylase deficiency. The case series report included 9 patients (6 male and 3 female) from 7 unrelated families who was identified novel and/or reported homozygous or compound heterozygous mutations in CYP11B1 gene. Diagnosed age was from 2 to 11 years old. All three female cases presented with ambiguous genitalia at birth. Other clinical features were hypertension (6/7 cases); hyperpigmentation (5/7 cases); pseudo-precocious puberty (male) (5/5 cases). Hypokalemia was noted in 3/7 cases. Three cases need antihypertensive drug associated with hydrocortisone replacement therapy. In conclusions, the clinical hallmark of 11β hydroxylase deficiency is variable and virilization and hypertension are the prominent clinical features of 11b hydroxylase deficiency. Biochemical identification of elevated precursor metabolites is not usually available and mutation analysis of CYP11B1 will held confirmation of diagnosis.

